# Mitochondrial Acetoacetyl-CoA Thiolase Deficiency: Three New Cases Detected by Newborn Screening Confirming the Significance of C4OH Elevation

**DOI:** 10.3390/ijns11030076

**Published:** 2025-09-06

**Authors:** Alessandra Vasco, Clarissa Berardo, Simona Lucchi, Laura Cappelletti, Giulio Tamburello, Salvatore Fazzone, Alessia Mauri, Francesca Fiumani, Diana Postorivo, Luisella Alberti, Michela Perrone Donnorso, Serena Gasperini, Francesca Furlan, Laura Fiori, Stephana Carelli, Laura Assunta Saielli, Cristina Montrasio, Cristina Cereda

**Affiliations:** 1 Center of Functional Genomics, Rare Diseases and Newborn Screening, Buzzi Children’s Hospital, 20154 Milan, Italy; alessandra.vasco@aorncaserta.it (A.V.); berardo.clarissa@asst-fbf-sacco.it (C.B.); simona.lucchi@asst-fbf-sacco.it (S.L.); cappelletti.laura@asst-fbf-sacco.it (L.C.); tamburello.giulio@asst-fbf-sacco.it (G.T.); salvatore.fazzone@asst-fbf-sacco.it (S.F.); alessia.mauri@unimi.it (A.M.); francesca.fiumani@unimi.it (F.F.); diana.postorivo@asst-fbf-sacco.it (D.P.); luisella.alberti@asst-ovestmi.it (L.A.); michelaperronedonnorso@gaslini.org (M.P.D.); stephana.carelli@asst-fbf-sacco.it (S.C.); laura.saielli@asst-fbf-sacco.it (L.A.S.); montrasio.cristina@asst-fbf-sacco.it (C.M.); 2 Pediatric Clinical Research Center “Romeo ed Enrica Invernizzi”, Department of Biomedical and Clinical Sciences, University of Milan, 20157 Milan, Italy; 3 Medical Genetics, Department of Health Sciences, University of Milan, 20146 Milan, Italy; 4 Pediatric Clinic, Endocrinology, Laboratory for the Study of Inborn Errors of Metabolism, Department of Neuroscience, Rehabilitation, Ophthalmology, Genetics, Maternal and Child Health, University of Genova, 16100 Genova, Italy; 5 Pediatric Rare Diseases Unit, Fondazione IRCCS San Gerardo dei Tintori, 20900 Monza, Italy; serena.gasperini@irccs-sangerardo.it; 6 Pediatric Intensive Care Unit, Fondazione IRCCS Cà Granda Ospedale Maggiore Policlinico, 20122 Milan, Italy; francesca.furlan@policlinico.mi.it; 7 Department of Pediatrics, Buzzi Children’s Hospital, 20154 Milan, Italy; laura.fiori@asst-fbf-sacco.it

**Keywords:** mitochondrial acetoacetyl-CoA thiolase deficiency, beta-ketothiolase deficiency, newborn screening (NBS), organic acids, organic acidemias (OAs), inborn errors of metabolism (IEMs), rare diseases

## Abstract

Acetoacetyl-CoA thiolase deficiency, also known as Beta-ketothiolase deficiency (BKTD), is an autosomal recessive organic aciduria included in the Italian newborn screening (NBS) panel. It is caused by mutations in the *ACAT1* gene, which encodes the mitochondrial acetyl-CoA acetyltransferase. Its deficiency impairs the degradation of isoleucine and acetoacetyl-CoA, leading to the accumulation of toxic metabolites. We describe three cases of BKTD. The first newborn showed increase in C5:1, C4DC/C5OH, C3DC/C4OH in the NBS. Urinary organic acids (uOAs) revealed marked excretion of 2-methyl-3-hydroxybutyrate. Tiglylglycine was absent. Genetic testing identified the compound heterozygosity for two pathogenic *ACAT1* variants. The second patient showed increased levels of C5:1, C4DC/C5OH, C3DC/C4OH in the NBS. uOAs revealed 2-methyl-3-hydroxybutyrate and tiglylglycine. A homozygous VUS in *ACAT1* was identified. The third case showed elevation of C4DC/C5OH, C3DC/C4OH in the NBS, with a slight increase in C5:1. uOAs showed 2-methyl-3-hydroxybutyrate and tiglylglycine. A homozygous missense VUS was identified in the *ACAT1* gene. BKTD exhibited variable NBS biochemical phenotypes across the three cases. While C5OH and C5:1, the primary markers, were not consistently elevated in all our cases, C4OH strongly increased in all three. Our findings support the use of C4OH in a combined marker strategy to improve BKTD NBS.

## 1. Introduction

Mitochondrial acetoacetyl-CoA thiolase deficiency (MIM #203750), also known as beta-ketothiolase deficiency (BKTD), is an inborn error of isoleucine catabolism characterized by urinary excretion of isoleucine catabolic intermediates, such as 2-methyl-3-hydroxybutyrate (2M3HB), 2-methylacetoacetate (2MAA), and tiglylglycine (TG) [1]. It is an autosomal recessive disorder, caused by homozygous or compound heterozygous variations in the acetyltransferase-1 gene (*ACAT1*; OMIM #607809) located on chromosome 11q22.3. The *ACAT1* gene encodes the mitochondrial acetoacetyl-CoA thiolase, which is a ubiquitous and important enzyme for isoleucine degradation [2]. This condition also impairs the body’s ability to process ketones, which are molecules produced during the breakdown of fats, which are important energy sources, particularly for the brain [1]. Affected children are typically asymptomatic at birth, with clinical manifestations usually emerging between five months and two years of age which include intermittent episodes of ketoacidosis, characterized by vomiting, dehydration, difficulty breathing, extreme lethargy, and occasionally, seizures until coma [3]. Metabolic stroke is another finding that has been increasingly reported in children with this condition [1]. Lactic acidosis with elevated ketones and sometimes hyperammonemia is frequently triggered by infections, fasting periods or increased intake of protein-rich foods. Episode frequency decreases with age, eventually stopping before adolescence. In between episodes, patients are often asymptomatic [1].

BKTD has been included in newborn screening (NBS) programs in many countries, including Italy since 2016 (Ministerial Decree of 13 October, https://www.gazzettaufficiale.it/eli/id/2016/11/15/16A08059/sg, accessed on 30 May 2025). The primary screening markers are 3-hydroxyisovalerylcarnitine (C5OH) and tiglylcarnitine (C5:1), even though some variability in biochemical presentation makes NBS for this rare disorder challenging [3]. Despite advances in tandem mass spectrometry, this disorder is known to yield a relatively high rate of false negatives [4]. We report three cases affected by BKTD that were identified early through NBS using FIA-MS/MS (first-tier test, 1TT) for the analysis of acylcarnitines and LC-MS/MS (second-tier test, 2TT) for the analysis of more specific biomarkers, promptly referred to the clinical reference center (CRC) for diagnosis and clinical follow-up [5]. Urinary organic acids (uOAs) performed on GC-MS were used as biochemical confirmation testing for the following biomarkers: 2M3HB, TG, and 2MAA; however, due to its instability, 2MAA is difficult to detect by GC-MS especially in non-fresh urine samples. In addition, next generation sequencing (NGS) was carried out on dried blood spots (DBSs) to analyze a virtual panel of 112 genes related to inborn errors of metabolism (IEMs), including the *ACAT1* gene (NM_000019.4). Genetic diagnosis has been confirmed by CRCs. All the procedures were performed as previously described [5,6].

## 2. Case Reports

### 2.1. Case 1

**Figure 1:**
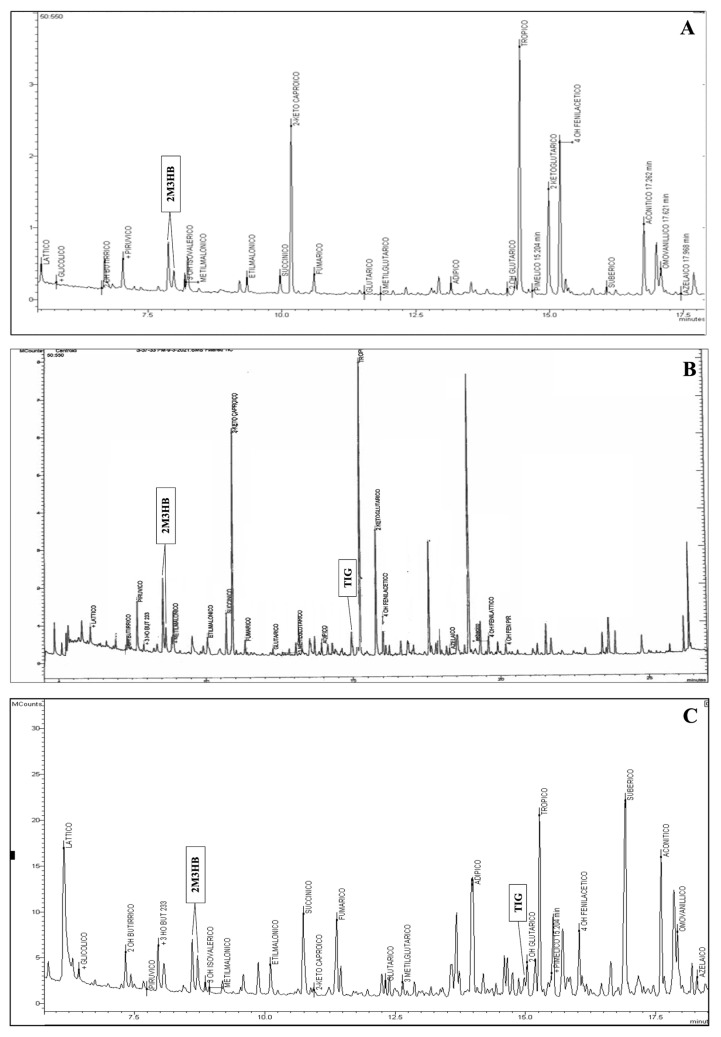
Chromatograms of urinary organic acids of the three cases showing the presence of: a peak corresponding to 2-methyl-3-hydroxybutyric acid (**A**); a peak corresponding to 2-methyl-3-hydroxybutyric acid and to tiglylglicine (**B**); a peak corresponding to 2-methyl-3-hydroxybutyric acid and to tiglylglicine (**C**).

In 2019, a woman gave birth to a full-term female infant weighing 2830 g via vaginal delivery. NBS revealed an increased concentration of C5:1 (0.15 µM; cut-off 0.03 µM), C4DC/C5OH (1.01 µM; cut-off 0.43 µM), and C3DC/C4OH (3.99 µM; cut-off 0.50 µM). At that time, the 2TT for BKTD was unavailable in our laboratory. A laboratory developed LC-MS/MS-based 2TT was applied retrospectively on the first DBS showing the presence of TG and 2M3HB (qualitative evaluation), as well as a significant increase in both D- and L-C4OH enantiomers, and the absence of their resolved isobaric C3DC (qualitative evaluation). The diagnostic suspect was confirmed by uOA analysis, which showed significant excretion of 2M3HB (Figure 1A), whereas TG was not detected up to the fifth month of life. The genetic analysis identified two pathogenic (P) variants in the *ACAT1* gene in compound heterozygosity, according to ACMG criteria [7]. The first variant, c.417delT (p.Met141Cysfs*7) in exon 5, is a frameshift mutation resulting from a single nucleotide deletion, not previously described. The second variant, c.472A>G (p.Asn158Asp; rs148639841) in exon 6, was reported in 3 ClinVar submissions [8,9] (Table 1).

The neonate was promptly referred to the CRC of Fondazione IRCCS Cà Granda Ospedale Maggiore Policlinico (Milan, Italy) for clinical evaluation. Segregation in parents has been performed using Sanger sequencing. Analysis of enzyme activity confirmed the deficiency (<1 nmol/(min·mg) with normal values equal to 9.00–30.30). A diet with controlled lipid and protein intake, along with the prevention of prolonged fasting through feeds approximately every three h during the first month of life, was implemented. Carnitine supplementation was initiated. Brain ultrasonography and echocardiography revealed no abnormalities. The patient’s medical history includes a single episode of metabolic decompensation associated with ketonuria during Sapovirus-induced gastroenteritis, which was managed with a 10% glucose intravenous infusion. At the most recent evaluation, at five years of age, clinical examination was unremarkable, with weight and height at the 50th percentile and psychomotor development within normal limits.

### 2.2. Case 2

The second case was a full-term male of 3810 g born by vaginal delivery from distant related parents. NBS revealed elevated levels of C5:1 (0.07 µM), C4DC/C5OH (0.74 µM), and C3DC/C4OH (3.3 µM). The application of 2TT showed the presence of TG, 2M3HB and a significant increase in both D- and L-C4OH enantiomers, and the absence of C3DC. A uOAs test showed a consistent presence of TG and 2M3HB, confirming diagnostic suspicion (Figure 1B). Genetic analysis identified the homozygous missense variant c.983C>T (p.Ala328Val; rs1057517702) in exon 10 of the *ACAT1* gene, classified as a variant of uncertain significance (VUS) according to ACMG criteria [7] and as reported in two ClinVar submissions (Table 1). Allele variation has been found in the general population with a frequency of 8.2 × 10^−7^, as reported in gnomAD, and there are no reported homozygous individuals in gnomAD. Computational evidence supporting a pathogenic effect of the variant includes a CADD score of 26.0 (>20 damaging) and a REVEL score of 0.896 (range 0–1, with higher scores reflecting greater likelihood of pathogenicity).

The neonate was admitted to the Neonatal Intensive Care Unit of the Metabolic Centre of Fondazione IRCCS San Gerardo dei Tintori (Monza, Italy) at four days of life in good clinical condition. Oral carnitine supplementation was initiated and breastfeeding was continued. A rigorous follow-up protocol was started, and the diagnosis was subsequently confirmed by molecular analysis. Management included age-appropriate avoidance of fasting and close monitoring during episodes of gastroenteritis or other catabolic stressors. Owing to early diagnosis and prompt initiation of treatment, the child—now four years old—has achieved normal psychomotor development and has not experienced any episodes of metabolic decompensation.

### 2.3. Case 3

Patient three was a full-term male of 3460 g born by vaginal delivery. NBS revealed elevated levels of C5:1 (0.07 µM), C4DC/C5OH (0.62 µM), and C3DC/C4OH (2.47 µM). The 2TT showed the presence of 2M3HB and an important increase both D- and L-C4OH enantiomers, and the absence of C3DC. uOAs analysis revealed significant excretion of 2M3HB and the presence of traces of TG, confirming the suspected diagnosis (Figure 1C). Genetic testing identified homozygosity for a missense variant in the disease gene, c.764A>T (p.Glu255Val) in exon 8, which has not been previously reported (Table 1). Computational evidence supporting a pathogenic effect of the variant includes a CADD score of 32.0 (>20 damaging) and a REVEL score of 0.783 (range 0–1, with higher scores reflecting greater likelihood of pathogenicity). The neonate was admitted to the Metabolic Department of Vittore Buzzi Children’s Hospital (Milan, Italy) at six days of life in good general health. Given the clinical suspicion of BKTD, a tailored dietary regimen was initiated, including feeds every three h with controlled caloric and protein intake. Oral carnitine supplementation was implemented. The patient was subsequently monitored for plasma ketone bodies and blood glucose levels, which remained undetectable and within the normal range, respectively, over the following weeks. The *ACAT*1 gene molecular analysis confirmed the diagnostic suspicion. As of the latest follow-up at 16 months of age, the child demonstrates normal psychomotor development and age-appropriate growth parameters.

## 3. Discussion

Clinical and biochemical diagnosis of BKTD may be challenging, as symptoms are often absent at birth and may emerge only several months later [1]. For this reason, NBS represents a useful approach for the early identification of this condition, preventing serious metabolic crises. The calculated incidence in the Lombardy (Italy) region was approximately 1:204,198, in accordance with the data reported in the literature [8]. C5OH and C5:1 acylcarnitines are the primary markers used in NBS for BKTD; however, even if only a slight increase in these classical markers is observed, a significant elevation of C4OH should always raise suspicion of BKTD [9]. In fact, among our cases, the use of 2TT enabled a differential diagnosis, effectively excluding malonic aciduria in the two cases with slight elevations of both C5:1 and C4DC/C5OH, but with a notable increase in C3DC/C4OH. Furthermore, in all BKTD cases, the presence of 2M3HB in the urine further corroborated the diagnostic suspicion.

It is noteworthy that, in all three cases C4OH was the most elevated marker in the NBS, in accordance with Soaters et al. which reported that D-3-hydroxybutyrate ketone bodies, accumulating in BKTD, can be converted into D-3-hydroxybutyrylcarnitine (C4OH) [10]. Therefore, as also reported by Lin et al., C4OH should be a screening marker alongside C5OH and C5:1 in a combined screening strategy to enhance the overall performance of BKTD detection and reduce the rate of false-negative results [4], since C4OH could increase in other diseases.

BKTD is an autosomal recessive disorder and variants in the *ACAT1* disease gene were detected in compound heterozygosity or homozygosity in all cases. Genetically, the first case also follows a classical pattern, presenting with pathogenic variants. The c.417delT was a frameshift mutation resulting from a single nucleotide deletion and led to a premature stop codon after seven amino acids, therefore it might be classified as pathogenic. The second variant c.472A>G, which affected a highly conserved amino acid, has been previously described in association with BKTD when found in combination with other disease-causing *ACAT1* variants [11,12]. In silico analysis suggests that this variant may be deleterious to the protein’s structure and function.

The other two cases showed homozygous variants both classified as VUS, of which one was already reported and the other one not. Despite the lack of functional studies to support the pathogenicity of the variations, we reasonably hypothesize that they could be causative of the disorder for several reasons. First, both variants were identified in the main disease-associated gene, consistent with autosomal recessive conditions. Second, both are missense variations, which aligns with the typical mutational spectrum of BKTD, as most pathogenic *ACAT1* variants reported in the literature are of the missense variation. Notably, disease-associated variants in *ACAT1* have been identified across all exons, with a higher concentration from exon 5 onward, particularly in exons 6 and 11 [12]. Third, the allele frequencies of these variants are extremely low across public databases, and in silico prediction tools suggest a deleterious effect. Moreover, for the c.764A>T variant, although previously unreported, two other distinct amino acid substitutions at the same codon, p.Glu255Asp (rs794727893) and p.Glu255Ala (rs1591370252) [12], have been described in association with the disease, further supporting its potential pathogenicity, as described by the PM5 ACMG criteria. It has been reported that most disease-associated *ACAT1* variants are “private”, having been reported in only a single family [12]. Consistently with this, we report that this variation was inherited from parents originating from Sardinian, a genetically isolated island considering the basis of classical autosomal markers, uniparental markers, and elevated linkage disequilibrium [13].

## 4. Conclusions

In conclusion, this study describes three newly identified cases of this rare metabolic disorder, underlining the value of expanding the number of reported cases to gain deeper insight into its biochemical phenotype. Our findings also support the relevance of C4OH as a useful biomarker in the context of newborn screening, integrated with an appropriated 2TT, which may facilitate earlier diagnosis and timely intervention.

## 5. Patents

Perrone Donnorso M., Cassanello M., Cereda C. A method and a kit for multiplexed second tier test application in Newborn Screening. Pending Patent application n. 102024000022824, on 14 October 2024.

## Figures and Tables

**Table 1:** Biochemical and genetic results of the three cases described.

Test	Analytes (µM; Cut-Off)	Case 1	Case 2	Case 3
Newborn screening				
1TT	C5:1 (0.03)	0.15 ↑↑	0.07 ↑	0.07 ↑
	C4DC/C5OH (0.43)	1.01 ↑	0.74 ↑	0.62 ↑
	C3DC/C4OH (0.50)	3.99 ↑↑	3.30 ↑↑	2.47 ↑↑
2TT **	C4OH	n.a. *	present ↑↑	present ↑↑
	2M3HB	n.a. *	present	present
	TG	n.a. *	present	*not present*
Biochemical and genetic confirmation test				
uOAs	2M3HB	present	present	present
	TG	*not present*	present	traces
NGS	Variant 1	c.417delT p.Met141Cysfs*7 (P)	c.983C>T p.Ala328Val(VUS) rs1057517702	c.764A>T p.Glu255Val(VUS)
	Variant 2	c.472A>G p.Asn158Asp (P) rs148639841		

1TT = first-tier test; 2TT = second-tier test; uOAs = urinary organic acids; NGS = next generation sequencing; C5:1 = tiglylcarnitine; C4DC = methylmalonilcarnitine; C5OH = 3-hydroxyisovalerylcarnitine; C3DC = malonylcarnitine; C4OH = 3-hydroxybutyrylcarnitine; 2M3HB = 2-methyl-3-hydroxybutyrate; TG = tiglylglycine; P = pathogenic; VUS = variant of uncertain significance; ↑ = increase; ↑↑ = strong increase; * the 2TT was performed retrospectively and not at the moment of NBS, showing the presence of TIG, 2M3HB and both D- and L-C4OH enantiomers. ** The 2TT was performed by extracting DBSs with an acidified aqueous solution containing internal standards and a reducing agent, incubating at 37 °C for 25 min and analyzing the resulting supernatant by UHPLC-MS/MS [5].

## Data Availability

Data are unavailable due to privacy.

## Abbreviations

The following abbreviations are used in this manuscript:

BKTD	beta-ketothiolase deficiency
NBS	newborn screening
ACAT1	acetyl-CoA acetyltransferase
VUS	variant of uncertain significance
2M3HB	2-methyl-3-hydroxybutyrate
2MAA	2-methylacetoacetate
TG	tiglylglycine
1TT	first tier-test
2TT	second tier-test
P	pathogenic variant
LP	likely pathogenic variant

## References

[1] Sass J.O., Fukao T., Mitchell G.A. (2018). Inborn Errors of Ketone Body Metabolism and Transport: An Update for the Clinic and for Clinical Laboratories. J. Inborn Errors Metab. Screen..

[2] Fukao T., Yamaguchi S., Kano M., Orii T., Fujiki Y., Osumi T., Hashimoto T. (1990). Molecular Cloning and Sequence of the Complementary DNA Encoding Human Mitochondrial Acetoacetyl-Coenzyme A Thiolase and Study of the Variant Enzymes in Cultured Fibroblasts from Patients with 3-Ketothiolase Deficiency. J. Clin. Investig..

[3] Fukao T., Sasai H., Aoyama Y., Otsuka H., Ago Y., Matsumoto H., Abdelkreem E. (2019). Recent Advances in Understanding Beta-Ketothiolase (Mitochondrial Acetoacetyl-CoA Thiolase, T2) Deficiency. J. Hum. Genet..

[4] Lin Y., Yang Z., Yang C., Hu H., He H., Niu T., Liu M., Wang D., Sun Y., Shen Y. (2021). C4OH Is a Potential Newborn Screening Marker—A Multicenter Retrospective Study of Patients with Beta-Ketothiolase Deficiency in China. Orphanet J. Rare Dis..

[5] Berardo C., Vasco A., Mauri A., Lucchi S., Cappelletti L., Saielli L., Rizzetto M., Biganzoli D., Montrasio C., Postorivo D. (2025). Expanded Newborn Screening in Italy: The First Report of Lombardy Region. Int. J. Neonatal Screen..

[6] Mauri A., Berardo C., Biganzoli D., Meta A., Benedetti S., Rey F., Messa L., Zuccotti G.V., Carelli S., Alberti L. (2024). Towards Genomic-Newborn Screening: Technical Feasibility of Exome Sequencing Starting from Dried Blood Spots. Mol. Genet. Metab. Rep..

[7] Richards S., Aziz N., Bale S., Bick D., Das S., Gastier-Foster J., Grody W.W., Hegde M., Lyon E., Spector E. (2015). Standards and Guidelines for the Interpretation of Sequence Variants: A Joint Consensus Recommendation of the American College of Medical Genetics and Genomics and the Association for Molecular Pathology. Genet. Med..

[8] Fukao T. (2004). Beta-Ketothiolase Deficiency.

[9] Duque Lasio M.L., Zaitsev M., Hobert J.A., De Biase I., Pasquali M., Yuzyuk T. (2025). C4OH-carnitine: An important marker of ketosis in patients with and without inborn errors of metabolism. Mol. Genet. Metab..

[10] Soeters M.R., Serlie M.J., Sauerwein H.P., Duran M., Ruiter J.P., Kulik W., Ackermans M.T., Minkler P.E., Hoppel C.L., Wanders R.J.A. (2012). Characterization of D-3-Hydroxybutyrylcarnitine (Ketocarnitine): An Identified Ketosis-Induced Metabolite. Metabolism.

[11] Zhen X.M., Twigg S.M., Wu T., Tabet E., McGill M.J., Constantino M., Mallawaarachchi A., Luo C., Thillainadesan S., Rahman Y. (2024). Diabetic Ketoacidosis in an Adult with Beta-Ketothiolase Deficiency (BKD) Involving a Novel *ACAT1* Variant: First Report of Established Diabetes in BKD and a Review of the Literature. Clin. Diabetes Endocrinol..

[12] Abdelkreem E., Harijan R.K., Yamaguchi S., Wierenga R.K., Fukao T. (2019). Mutation Update on *ACAT1* Variants Associated with Mitochondrial acetoacetyl-CoA Thiolase (T2) Deficiency. Hum. Mutat..

[13] Chiang C.W.K., Marcus J.H., Sidore C., Biddanda A., Al-Asadi H., Zoledziewska M., Pitzalis M., Busonero F., Maschio A., Pistis G. (2018). Genomic History of the Sardinian Population. Nat. Genet..

